# Characterizing Cell Stress and GRP78 in Glioma to Enhance Tumor Treatment

**DOI:** 10.3389/fonc.2020.608911

**Published:** 2020-12-11

**Authors:** Kristie Liu, Kathleen Tsung, Frank J. Attenello

**Affiliations:** ^1^ Keck School of Medicine of the University of Southern California, Los Angeles, CA, United States; ^2^ Department of Neurosurgery, Keck School of Medicine of the University of Southern California, Los Angeles, CA, United States

**Keywords:** GBM—Glioblastoma multiforme, UPR—unfolded protein response, glioma, GRP94, ER stress, TMZ (Temozolomide), glucose regulated protein 78 (GRP78), GBM therapeutic target

## Abstract

Glioblastoma (GBM) is the most common primary brain tumor, carrying a very poor prognosis, with median overall survival at about 12 to 15 months despite surgical resection, chemotherapy with temozolomide (TMZ), and radiation therapy. GBM recurs in the vast majority of patients, with recurrent tumors commonly displaying increase in resistance to standard of care chemotherapy, TMZ, as well as radiotherapy. One of the most commonly cited mechanisms of chemotherapeutic and radio-resistance occurs *via* the glucose-regulated protein 78 (GRP78), a well-studied mediator of the unfolded protein response (UPR), that has also demonstrated potential as a biomarker in GBM. Overexpression of GRP78 has been directly correlated with malignant tumor characteristics, including higher tumor grade, cellular proliferation, migration, invasion, poorer responses to TMZ and radiation therapy, and poorer patient outcomes. GRP78 expression is also higher in GBM tumor cells upon recurrence. Meanwhile, knockdown or suppression of GRP78 has been shown to sensitize cells to TMZ and radiation therapy. In light of these findings, various novel developing therapies are targeting GRP78 as monotherapies, combination therapies that enhance the effects of TMZ and radiation therapy, and as treatment delivery modalities. In this review, we delineate the mechanisms by which GRP78 has been noted to specifically modulate glioblastoma behavior and discuss current developing therapies involving GRP78 in GBM. While further research is necessary to translate these developing therapies into clinical settings, GRP78-based therapies hold promise in improving current standard-of-care GBM therapy and may ultimately lead to improved patient outcomes.

## Introduction

### Gliomas: Background

Gliomas are the most commonly diagnosed primary neoplasms in the central nervous system, constituting up to 81% of malignant brain tumors (1). Malignant gliomas include anaplastic astrocytoma (grade 3) and glioblastoma multiforme (GBM, grade 4). GBM is the most common histology, comprising 45% of gliomas (1), with a prognosis that remains dismal, with median overall survival for GBM at about 12 to 15 months (2) and a 5-year survival of only 5.5% despite therapy (3). The incidence of GBM also increases with age, and is associated with devastating neurological effects, including weakness, visual and sensory changes, headaches, seizures, and alterations in mood, memory, or executive function (4).

### Current Management

Standard of care for a newly suspected GBM involves surgical resection (or biopsy), followed by chemotherapy and radiation therapy. The standard chemotherapeutic regimen utilizes temozolomide (TMZ), a well-tolerated drug that has been shown to delay tumor progression and provide modest improvement in patient survival (5). Specifically, TMZ exerts anti-tumor effect *via* DNA methylation and substitution of cytosine by thymine (6). This repeated substitution activates the mismatch repair mechanism, which triggers cell stress and apoptosis in response to the detection of recurrent errors in DNA.

### Treatment Limitations and Resistance

Despite surgery, chemotherapy, and radiotherapy, the majority of GBM patients experience tumor recurrence with increased chemo- and radio-resistance. Furthermore, there is currently no standard of care in second line management following initial adjuvant treatment (2). Because the mortality of GBM remains high and tumor chemo- and radio-resistance remain a critical challenge, new treatment modalities or approaches are needed to improve outcomes. Such treatment strategies have included multiple chemotherapeutic agents, anti-angiogenic therapy, and immunotherapy. Several promising treatments have focused on the unfolded protein response (UPR), a cellular stress response to accumulated proteins in the lumen of the endoplasmic reticulum (ER) (7). The UPR has emerged as one of the more promising targets due to its role in tumor survival and therapeutic resistance. Specifically, glucose-regulated protein 78 (GRP78) has emerged as a potential target in the majority of these studies due to its role as a central modulator of the UPR. Overexpression of GRP78 has been repeatedly demonstrated to modulate malignant and aggressive phenotypes in GBM tumor cells (8–12). In addition, GRP78 expression has been noted to promote propagation of glioma stem cells (GSCs), tumor-replenishing cells that form the pool of the highly proliferating transient cell population, while also driving GBM resistance and recurrence (13, 14). While UPR and GRP78 data is abundant in the literature, a focus on the role of this system in GBM is limited. Here, we have provided an updated review, including several years of novel studies evaluating and targeting endoplasmic reticulum proteostasis in GBM (15), with emphasis on the significance of GRP78 and targeted therapies for GRP78. We specifically aim to summarize the literature assessing the role of GRP78 and other mediators of the UPR within GBM, including novel studies exploring the role of the UPR in glioma stem cells. Furthermore, we review developing GBM therapies and treatment delivery methods to demonstrate how GRP78 is a compelling therapeutic target and biomarker that could potentially translate to improved GBM therapy and care.

## GRP78 and the Unfolded Protein Response

### What Is GRP78?

The UPR is a cellular stress response that is activated when unfolded or misfolded proteins accumulate in the lumen of the endoplasmic reticulum. GRP78, also known as immunoglobulin heavy chain binding protein (BiP), is a well-studied chaperone heat shock protein that is central to the modulation of the UPR. The GRP78 protein primarily resides in the lumen of the ER but can be found on the ER membrane and on the cell surface (16, 17). As a molecular chaperone, GRP78 is important for protein folding and assembly, binding calcium in the ER, and export of misfolded proteins for degradation (18). GRP78 has two domains modulating UPR function, a nucleotide-binding domain (NBD) and a substrate binding domain (SBD) (19), described in further detail below. GRP78 expression is directly correlated with ER activity and regulated on the transcriptional level, based on an analysis of the human GRP78 gene demonstrating that the GRP78 promoter contains an ER stress-sensitive region located 170 nucleotides upstream from the transcription initiation site (20).

### GRP78's Role in the UPR

**Figure 1:**
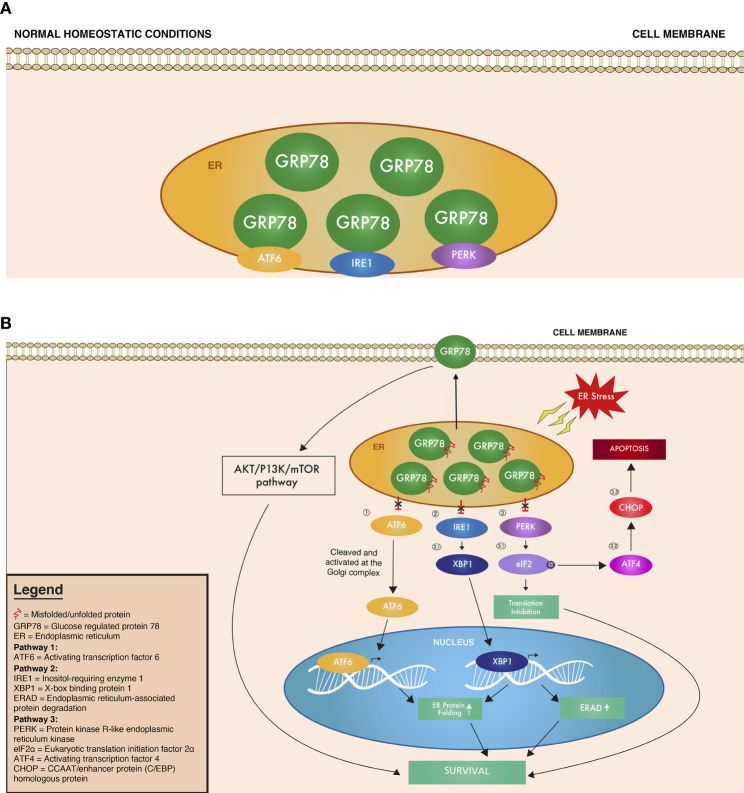
Overview of the UPR signaling cascade under **(A)** normal homeostatic conditions where GRP78 is bound to ATF6, IRE1, and PERK, inhibiting their function and **(B)** ER stress conditions, where ATF6, IRE1, and PERK are no longer bound, and thus no longer inhibited by GRP78. Circled numbers refer to the corresponding protein in the text and in **Table 1**.

In the UPR, GRP78 functions as a molecular chaperone that binds to misfolded proteins and unassembled complexes and initiates ER-associated degradation (ERAD). ERAD targets these misfolded proteins for degradation *via* ubiquitination and proteasomal degradation. In normal homeostatic cell conditions, GRP78 is in an inactive form bound to (1) activating transcription factor 6 (ATF6), (2) protein kinase RNA-like endoplasmic reticulum kinase (PERK), and (3) inositol-requiring enzyme 1 (IRE1), all of which are UPR transmembrane sensors of cellular stress (**Figure 1A**, **Table 1**) (24). When unfolded proteins accumulate in the ER, GRP78 is released from these UPR stress sensors to exert its various aforementioned functions (**Figure 1B**, **Table 1**).

**Table 1:** Overview of the UPR signaling cascade under normal homeostatic conditions and ER stress.

Figure Reference	UPR Element	Normal Cell Conditions	ER Stress Conditions	References
	GRP78	Bound to ATF6, IRE1, PERK	GRP78 proteins bind misfolded proteins and prevent their transport, correct protein folding, and bind to ER calcium. Under stress, a subset of GRP78 translocates to the surface -> PI3K/Akt/mTOR pathway	Surface GRP78 (21–23), GRP78 (18, 24)
1	ATF6	Bound to GRP78	Disinhibited by release from GRP78 -> cleaved and activated at Golgi apparatus -> moves to nucleus as active transcription factor that upregulates proteins that promotes ER protein folding	(18, 25, 26)
2	IRE1	Bound to GRP78	Disinhibited by release from GRP78 -> endoribonuclease activity breaks mRNA intron -> encodes XBP1	(18, 27)
2.1	XBP1	Not transcribed	Encoded by IRE1 -> targets genes in protein folding and ERAD	(18, 27)
3	PERK	Bound to GRP78	Disinhibited by release from GRP78 -> phosphorylates eIF2α	(18, 28)
3.1	eIF2α	Unphosphorylated	Activated by phosphorylation -> inhibits initiation of translation -> decreases protein synthesis and protein influx in the ER; Activated by phosphorylation -> activates ATF4	(18, 28)
3.2	ATF4	Inactivated	Activated by eIF2α -> activates CHOP	(25, 29)
3.3	CHOP	Inactivated	Activated by ATF4 -> apoptosis	(25, 29)

Figure Reference numbers correspond with **Figure 1** and the text.

### Downstream Effectors of the UPR

Upon release from GRP78, the UPR stress sensors (1) ATF6, (2) IRE1, and (3) PERK are also released to create a cascade of signaling down interacting UPR pathways (**Figure 1B**, **Table 1**). (1) ATF6 released from GRP78 then translocates to the Golgi apparatus, where it is cleaved into its active form. This active ATF6 then moves to the nucleus as a transcription factor that upregulates proteins involved in increasing the folding capacity of the ER (18). Meanwhile, after its release from GRP78, (2) the active form of IRE1 exerts its endoribonuclease activity, breaking a segment of mRNA intron and encoding X-box binding protein 1 (XBP1), which targets genes responsible for protein folding and ERAD (18, 27). Finally, when (3) PERK is released and activated, it phosphorylates the α subunit of eukaryotic translation initiation factor (eIF2α), which inhibits the initiation of translation, thus decreasing protein synthesis and the influx of proteins into the ER (18, 28). The phosphorylated eIF2α also activates activating transcription factor 4 (ATF4), which, in turn, activates the CCAAT/enhancer binding protein homologous transcription factor (CHOP/GADD153), which is associated with apoptosis (25, 29). All of these cascades continue to activate molecular targets to either promote survival of the cell or apoptosis, depending on the level of ER stress detected. If homeostasis cannot be restored, the pro-apoptotic arm of the UPR is induced, which is represented by the induction of CHOP (18, 24). CHOP then suppresses antiapoptotic outer mitochondrial membrane protein BCL2 and induces proapoptotic proteins, including death receptor 5 (DR5) and ER oxidoreductin protein 1α. All of the ATF6, IRE1, and PERK pathways overlap and with each pathway involved in both pro-survival and pro-apoptosis cellular responses. The mechanisms and nuances behind the coordinated activation of pro-survival or pro-apoptosis pathways still remain unclear, and further research is needed to pinpoint which ER stress signals can trigger apoptosis over survival and *vice versa*.

## GRP78 in Glioblastoma and its Role in Resistance

### GRP78 in GBM *vs.* Normal Tissue

Because the UPR manages cellular stress, it is a central component in promoting tumor survival. Solid tumors are, by definition, inherently stressed cells as a result of dysregulated cell proliferation, which leads to inadequate blood supply, hypoxia, nutrient deprivation, and immune reactions (30). To adapt to these conditions, tumors upregulate multiple pathways promoting cell survival, including those involved in protein folding and stabilization. These survival pathways subsequently upregulate molecular chaperone proteins and heat shock proteins in the UPR (30–32). This behavior is prevalent among multiple tumor types including GBM, as elevated GRP78 levels in gliomas compared to normal human astrocytes have been repeatedly demonstrated and confirmed *via* immunohistochemistry, immunoblotting, and proteomic approaches in patient samples (10, 33, 34). These data suggest a chronic activation of the UPR and GRP78 activity within GBM cells. While differential GRP78 expression has been seen between GBM tumors and normal astrocytes, very limited data exists on differential GRP78 expression across core and leading edge of GBM tumors as well as their surrounding parenchyma. While available data from tumor microarrays have included multiple samplings from single tumors, no clear data exist regarding differential expression from surrounding cortical tissue (35). A potential direction for future studies may potentially compare GRP78 expression in GBM tumor cores, leading edge, surrounding T2 positive signal, and surrounding cortex to better characterize the role of GRP78 in modulating tumor, invasion and microenvironment.

### GRP78 Correlates With Malignancy (Grade, Proliferation, Migration)

In addition to upregulation within tumor cells, several studies on clinical tumor samples have shown that GRP78 is associated with patient tumor phenotype and behavior. GRP78 expression is notably higher in more proliferative GBM cell lines, while both RNA and protein expression have been found to increase with tumor grades in patient astrocytoma specimens (8–12). When comparing GBM cell lines, cell lines with higher basal cell proliferation and migration have up to three-fold higher GRP78 expression than those cell lines with less aggressive phenotypes (36). Overexpression of GRP78 was also found to limit tumor cell apoptosis, with decreased activation of pro-apoptotic caspase 7 signaling (11). Modulation of GRP78 *via* siRNA knockdown has further resulted in decreased proliferation of GBM cell lines while attenuating pro-survival pathways, namely Akt and ERK1/2, suggesting these pathways may at least partially mediate the effects of GRP78 on GBM cell proliferation (8). Patient outcomes are linked to such tumor progression, with more rapidly preoperative tumor growth rates in glioblastoma resulting in increased tumor size and more limited extent of tumor resection (37). As noted above, increasing expression level of GRP78 is strongly correlated with increasing tumor grade and malignancy, with grade IV glioblastoma (and therefore those tumors with poorest prognosis) expressing the highest levels of GRP78. Patterns of GRP78 are also correlated with grade. GBM is associated with a scattered pattern, while grade I astrocytoma and non-neoplastic tissue are associated with a highly grouped pattern (36). Because cellular and genetic heterogeneity is a hallmark of glioblastoma, studies have suggested the scattered GRP78 pattern noted in high grade gliomas relative to normal glial tissue/low grade tumors may contribute to the heterogeneity of high grade astrocytomas.

### GRP78 Modulates Chemotherapy and Radiotherapy Responses

Various studies on GRP78 have also demonstrated that GRP78 has an important role in recurrent GBM and tumor progression after initial treatment. Of particular importance is temozolomide (TMZ), the standard-of-care chemotherapeutic treatment for GBM. TMZ has been shown to result in activation of the UPR in GBM cells, inducing increased levels of UPR markers, GRP78 and CHOP (10). However, when repressing GRP78 in drug sensitivity analyses with colony survival assays, GBM cells are notably more sensitive to TMZ and multiple other chemotherapeutic agents including 5-FU, irinotecan, etoposide, and cisplatin (10, 11). Conversely, overexpression of GRP78 promotes GBM cell resistance to not only TMZ treatment (10), but also etoposide and cisplatin-induced apoptosis (11). Radiation therapy, also a critical component of first-line GBM adjuvant treatment, has shown induction of GRP78, with radiation-induced reactive oxygen species associated with induction of ER stress (26). Furthermore, the effects of radiation therapy are similarly modulated by GBM expression of GRP78. Radio-resistance of GSCs has been shown to increase with GRP78 expression (38), and knockdown of GRP78 sensitized cells to gamma radiation (11).

While GRP78 has been correlated with increasing glioma grade, as noted above, survival analyses among grade IV GBM patients have also noted direct correlations between aggressive clinical behavior and GRP expression. Specifically, GRP78 is elevated in patient GBM samples compared to non-neoplastic brain tissue, with the greatest levels of GRP78 in patients with shorter overall survival (11, 39). Furthermore, more rapid tumor recurrence (decreased time to progression) after initial tumor resection is associated with higher GRP78 levels (8). Importantly, direct clinical data evaluating primary and recurrent GBM tissue from patients following TMZ and radiation therapy demonstrates that GRP78 levels are elevated in resected GBM samples following these treatments (9). These studies may be of particular relevance to patient outcomes, with more aggressive phenotypes noted among patients with recurrent glioblastoma (40, 41). Taken together, clinical data reveal GRP78 expression not only increases tumor aggressiveness, but also shows upregulation in recurrent GBM in response to cytotoxic drugs, making GRP78 an attractive target for therapy as well as implicating it as a prognostic marker.

### Cell Surface GRP78

GRP78 predominantly resides in the ER lumen within normal cells, with the majority of GRP78 studies primarily focusing on cytosolic or total GRP78. However, in tumor microenvironments where GRP78 is overexpressed, GRP78 also localizes to the surface of GBM cell membranes (21). In studies across multiple tumor types, cell surface GRP78 (csGRP78) acts as a co-receptor and participates in various signaling processes, modulating tumor apoptosis, proliferation, and motility (22, 42). Furthermore, re-location of GRP78 to the cell surface is notably associated with drug resistance and cell transformation (23). Modulation of cell surface GRP78 in multiple tumor types has also shown effects on behaviors of cancer stem cell populations (43, 44). In one of the more well characterized pathways, surface GRP78 has been shown to interact with and promote activation of the tumorigenic PI3K/AKT pathway through complex formation with PI3K in prostate cancer (22).

More recently, cell surface GRP78 has been explored in the context of gliomas. Unsurprisingly, cell surface GRP78 has been detected in multiple high-grade glioma cell lines *via* immunocytochemistry and immunoblotting. Treatment of this surface GRP78 with a polyclonal antibody decreased high-grade glioma cell line survival and population growth (21). Notably, greater reductions in glioma growth are seen with antibody treatments of lower grade tumors. Combination treatment, with both radiotherapy and antibody to cell surface GRP78, also results in efficacy on tumor apoptosis relative to radiation alone (45). Targeting of cell surface GRP78 likely exerts apoptotic effects *via* the Akt and mTOR pathways, as antibody treatment of GBM cell lines revealed decreases in phosphorylated and total Akt and mTOR. Intravenous injection of antibody has proven a feasible delivery mechanism, as anti-GRP78 antibodies delivered *via* mouse tail vein are noted to extravasate cell from vasculature to tumor. Surface expression of GRP78 thus provides an accessible binding site for targeted GRP78 therapeutics discussed below. Although combination therapy of radiation and antibody to cell surface GRP78 has demonstrated promise in *in vitro* and in mouse xenograft models, Akt/mTOR pathway inhibitors have yet to demonstrate meaningful benefit in GBM clinical trials (46). While limitations have been attributed to the blood brain barrier, GBM tumor heterogeneity, and possible activation of alternative pathways that allow for immune escape, the lack of direct Akt/mTOR pathway inhibitors must be noted in subsequent studies. The expansion of studies to include *in vivo* intracranial GBM preclinical models, or evaluation of csGRP78 in clinical patient samples may shed further light on efficacy and mechanism of targeting csGRP78.

### Influence of Downstream GRP78 Elements on GBM Behavior

GRP78 ultimately modulates GBM phenotype *via* the well-characterized downstream elements in its pathway, with several studies showing direct correlation of these elements with GBM oncogenesis and resistance to treatments. As previously mentioned, these downstream elements include the UPR stress sensors (1), ATF6 (2), IRE1, and (3) PERK. The first of these downstream effector pathways, the (1) ATF6 pathway, was shown to contribute to GBM radiotherapy resistance, with ATF6 knockdown resulting in increased radiotherapy sensitivity. However, ATF6 activity does not entirely explain the above noted GRP78-modulated GBM phenotypes, as ATF6 expression was not shown to be correlated with astrocytoma tumor grade (25, 26). Meanwhile, *in vitro* knockdown and overexpression GBM studies have revealed the (2) IRE1 pathway to be involved in tumor growth, migration, invasion, and neovascularization in GBM (25, 47–50). Specifically, *in vitro* knockdown studies of IRE1 commonly modulate GBM expression of extracellular matrix proteins including SPARC/Osteonectin, a protein eliciting cell shape changes and modulating synthesis of extracellular matrix (47). IRE1 has specifically shown modulation of angiogenesis *via* studies showing that increased IRE1-XBP1 signaling stimulated angiogenesis, while decreased IRE1-XBP1 signaling suppressed angiogenesis (51). The (3) PERK pathway has been shown to initiate signaling that promotes protective metabolic processes such as glycolysis under cell stress (52), while also mediating autophagy responses to targeted GRP78 therapies such as OSU-03012, a treatment discussed in detail below (53). In evaluation of tissue microarrays from 148 glioblastoma patients, decreased expression of ATF4, a downstream element of the PERK pathway (as described above) is associated with prolonged overall survival (54). This was interpreted by the authors as clinical evidence, suggesting a link specifically between the PERK branch of the UPR and GBM patient prognosis. Interestingly, despite results implicating PERK and ATF4 in promotion of tumor survival, they have also shown upstream activation of pro-apoptotic CHOP, exemplifying how UPR pathways have roles in both pro-survival and pro-apoptotic responses.

Regulation of these pro- and anti-apoptotic pathways and their opposing consequences is complicated and continues to be studied. The pro-survival arm of endoplasmic reticulum stress, triggered by conditions of glucose starvation, hypoxia, Ca2+ depletion, and misfolding of proteins, initially activates GRPs and the UPR in an attempt to restore normal functioning (55). In this survival arm, GRP78 forms a complex with caspase-7, a mediator of apoptosis, inhibiting its activity. The pro-apoptotic arm seems to predominate when the protein aggregation is persistent and the stress is not resolved, which subsequently triggers signaling to become pro-apoptotic. CHOP, which is generally induced in ER stress, is thought to mediate the commitment to the pro-apoptotic response. When the stress is prolonged and severe, the PERK and IRE1 pathways converge on CHOP and increase its induction (56). Consequently, CHOP increases expression of pro-apoptotic genes and decreases expression of anti-apoptotic genes, including Bcl2. IRE1α also promotes both pro- and anti-apoptotic events including the activation of JNK and caspase pathways, as well as splicing of XBP-1 (57). Indeed, the interaction between UPR elements is very complex, with data from knockout of each individual UPR component suggesting that no single component is strictly necessary for ER induced apoptosis, underlining the need for further study (57).

### Glioma Stem Cells

Multiple UPR elements, including GRP78 and PERK, have been implicated in function of glioma stem cells (GSCs). GSCs are defined as a subpopulation of stem-like glioblastoma cells capable of self-renewal, differentiation into cortical lineages (astrocyte, oligodendrocyte, neuron), and have tumorigenic capacity. Not only are GSCs hypothesized to be the cell of origin for GBM, but the aggressive characteristics of GBM, including vascularization, invasion, chemo-resistance, radio-resistance, and recurrence, are often attributed to GSCs (58).

While few studies have evaluated UPR and GRP78 in GSCs, recent data suggests that GSCs show differential responses to cell stress, as UPR activation *via* a common cell stress agent, thapsigargin (Tg), results in variable results when comparing patient-derived GSCs (from mesenchymal and proneural subtypes) and their differentiated counterparts. GBM neurospheres show upregulation of all three branches of the UPR, with increased PERK phosphorylation, activation of IRE1α, and accumulation of ATF6. The contribution of apoptosis to cell death was seen with activation of caspases 3/7, while an apoptotic inhibitor suppressed cytotoxicity. Specifically, Tg-induced ER stress results in decreased survival of GSCs relative to effects on differentiated tumor cells (54). This GSC-specific sensitivity may be mediated by ER stress upregulation of the pro-apoptotic PERK signaling pathway of the UPR, as inhibition of PERK suppressed these GSC-specific cytotoxic effects. GSC self-renewal, in contrast to apoptosis, may be mediated by the PERK pathway. Specifically, cell stress was noted to decrease both as well as expression of Sox2, an essential protein in maintaining pluripotency and self-renewal properties of stem cells. These novel GSC effects are regulated by the PERK branch of the UPR during cell stress, as repression of the PERK branch (but not the IRE1/XBP1 branch) limits these effects (54). GSC self-renewal in association with PERK branch suppression has also been noted when BMI1, an epigenetic modifying factor, increases both GSC self-renewal and cell stress by suppressing ATF3, a downstream element of the PERK signaling pathway. Decreased ATF3 expression is also associated with increased stemness and poor prognoses (35, 59, 60). Finally, though the role of GSCs in GBM invasion has yet to be fully characterized, invasive GBM cells have been shown to have increased stemness, and future studies may explore whether invasive GBM cells beyond the tumor edge have more stem-like cells containing elevated GRP78 expression (57). Given the hypothesized central role of GSCs in GBM aggressiveness and resistance to therapy, induction of ER stress may offer selective targeting of the GSC subset. Ultimately, further studies are warranted to elucidate effects of UPR induction and GRP78 elevation on both GSC and differentiated GBM populations.

### Glucose-Regulated Protein 94

Although GRP78 is the more thoroughly investigated glucose-regulated protein in the UPR pathway, glucose-regulated protein 94 (GRP94) is another molecular chaperone, localized in the ER, that shares functions with GRP78. Similarly, GRP94 has been implicated in aggressive glioma behavior. Evaluation of GRP94 mRNA and protein levels reveal significant elevations in GRP94 in high-grade glioblastoma when compared with normal brain tissues (33). In addition, a gradual increase in GRP94 protein and RNA levels in patient samples of grade II to grade IV gliomas is also noted, with the highest levels of GRP94 in grade IV GBM. Just as with GRP78, high GRP94 levels when evaluated across 20 glioma patients were also associated with a significantly shorter overall patient survival. Functional evaluation of GRP94 *in vitro* reveals knockdown of GRP94 in GBM cell lines that suppressed cellular proliferation, impaired colony formation ability, and inhibited cell migration and invasion ability. However, microarray analysis of GRP94 knockdown cells suggests that, while similar to effects of GRP78, GRP94 may instead exert its effects *via* downstream dysregulation of the Wnt/β-catenin signaling pathway, which normally promotes the proliferation of GBM cells (61). Finally, there appears to be at least a partial interplay between GRP94 and GRP78 in GBM cells. Specifically, GRP94 expression appears to increase when GRP78 is suppressed, but not enough to completely compensate for its loss (18, 62). Thus, GRP94 may be a promising therapeutic target similar to GRP78, though it appears to function through different pathways. However, research on GRP94 is limited and more studies are warranted to further elucidate GRP94's role in GBM tumor malignancy.

### GRP78 in Tumor Vasculature

GRP78 may influence not only GBM tumor cells, but the surrounding microenvironmental vasculature. GRP78 is notably significantly elevated in both *in situ* and *in vitro* primary cultures of human brain endothelial cells derived from blood vessels of malignant glioma tissues. In contrast, there appears to be minimal GRP78 expression in normal brain tissues and blood vessels. Functional studies further support a critical role for GRP78 in GBM endothelial cells, as GRP78 knockdown increases tumor endothelial cell susceptibility to not only TMZ, but multiple chemotherapeutic agents, including CPT-11 and etoposide. Conversely, GRP78 overexpression results in increased chemo-resistance of tumor endothelial cells (63). Though studies on GRP78 in tumor vasculature in the context of GBM is limited, there has also been evidence from studies on other solid tumors that suggest an important regulatory role for GRP78 in tumor vasculature. In a transgene-induced mammary tumor model, interference with GRP78 function exhibited dramatic reduction in the microvessel density of endogenous mammary tumors with no effect on normal organs, and a follow-up study demonstrated that interference with GRP78 function suppressed tumor growth and angiogenesis during the early phase of tumor growth (64). This suggests that GRP78 has a critical role in regulation of vasculature specific to the tumor microenvironment, while limiting injury to normal vasculature. While such data suggest GRP78 and the UPR may represent a therapeutic target for not only GBM tumor but associated microvasculature, further study is necessary both *in vivo* and at a from patient samples to clarify safety of such an approach.

An accumulating abundance of evidence thus continues to support a significant role for GRPs in modulating glioma and GSC behavior, as well as chemotherapy resistance. Therapeutic resistance of GBM cells to therapy remains a critical barrier to improving patient survival. Multiple groups have therefore focused on GRPs as therapeutic targets, developing several promising GRP-targeted agents to improve sensitivity to current conventional therapies and improve clinical outcomes.

## GRPs as Therapeutic Targets

In light of the potential that targeting GRPs have in improving glioblastoma therapy, various studies have sought to inhibit GRP78 using various agents and to examine their potential to be used in conjunction with current conventional therapies and in clinical settings. Other therapies modulate GRP78 expression without direct interaction or utilize GRP78 in treatment delivery modalities. While some efforts have directly targeted GRP78 *via* rational drug design or identified efficacious GRP targeting *via* screening, other targeted GBM therapies have incidentally been found to show effect in part through GRP effects. These therapies include natural products, fusion proteins, antibody-based treatments, phage-based treatment delivery methods, and more, all of which are shown in **Table 2**. More details on GRP78 interactions and downstream signaling effects of these GRP78-based therapies are also outlined in **Table 3**. The majority of these studies have not yet reached clinical trials, but *in vitro* and *in vivo* studies show promise in decreasing aggressive tumor phenotype upon treatment and reducing resistance to chemotherapy and radiation therapy.

**Table 2:** General characteristics of GRP78-based treatments.

Treatment	Type	Target	Enhances effects of:	Combination therapy in GBM	Mechanism	Effect	Model	Clinical trials	References
**Epigallocatechin 3-gallate (EGCG)**	Natural product	GRP78 (NBD domain)	TMZ; Others: 5-fluorouracil taxol vinblastine gemcitabine and tumor necrosis factor (TNF)-related apoptosis-inducing ligand (TRAIL), doxorubicin, paclitaxel, interferon-α2b	TMZ + EGCG	Impairs GRP78 function	Enhanced cytotoxicity when used with TMZ (not as monotherapy)	Human cell lines, *in vivo*	No	(58, 65)
**Honokiol**	Natural product	GRP78 (NBD domain)	TMZ; Others: fenretinide, bortezomib	TMZ + honokiol	Interferes with GRP78 folding	Induced ER stress-mediated apoptosis +/− TMZ	Human cell lines	No	(66–68)
**OSU-03012**	Celecoxib derivative	GRP78 (NBD domain)	Radiotherapy	Radiotherapy + OSU-03012; GRP78 inhibition further enhances effects	PDK1 inhibitor, GRP78 inhibitor, PERK signaling	Enhanced radiosensitivity, prolonged survival	Human cell lines, *in vivo* mouse models	No	(65, 69)
**Celecoxib and bortemozib**	Celecoxib-based	ER stress	N/A	Celecoxib + bortezomib + GRP78 inhibition	Augment ER stress	Induced ER stress-mediated apoptosis	Human cell lines	No	(70)
**Perillyl alcohol (NEO100)**	Monoterpene	ER stress	TMZ; Others: DMC, nelfinavir	TMZ + NEO100	Disruption of survival pathways	Induced more apoptosis +/− TMZ, reduced GBM invasive capacity, prolonged survival	Human cell lines, *in vivo*	Yes	(71–73)
**HA15**	Small molecule inhibitor	GRP78	N/A	N/A	Binds and inhibits GRP78 and disrupts GRP78 complexes with UPR transmembrane stress sensors	Induction of apoptosis	Human cell lines, *in vivo* mouse models	No	(74, 75)
**IT-139**	Small molecule inhibitor	GRP78	Chemotherapy	N/A - not yet studied in GBM	Involves transcriptional and post-transcriptional mechanisms	Decreases therapeutic resistance	Human cell lines, *in vivo* human xenograft studies	No	(76)
**EGF-SubA**	Fusion protein	GRP78	TMZ and ionizing radiation	TMZ + radiation therapy + EGF-SubA	Cleaves GRP78	Delayed tumor growth, enhanced effects of TMZ and ionizing radiation	Human cell lines, *in vivo* mouse models	No	(77)
**Anti-GRP78 antibody**	Antibody-based	surface GRP78	Ionizing radiation	ionizing radiation + anti-GRP78 antibody	Suppression of PI3K/Akt/mTOR signaling	Enhanced effects of ionizing radiation, resulted in tumor delay	Human cell lines, *in vivo* mouse xenograft models	No	(45)
**RGD ligand-directed phage with GRP78 promoter**	Treatment delivery	GRP78	Expression of therapeutic transgenes	N/A	RGD tumor homing ligand and *GRP78* promoter	Improved expression of therapeutic transgenes compared to standard promotor phage	Human cell lines, *in vivo*	No	(78)
**TMZ-induced AAV phage with GRP78 promoter**	Treatment delivery	GRP78	TMZ, expression of therapeutic transgenes	TMZ + phage	RGD4C ligand binding, TMZ-induced GRP78 expression activates therapeutic genes with *GRP78* promoter	Permits dose reductions of TMZ	Human cell lines, mouse xenograft models	No	(79)
**GRP78-binding peptide (GIRLPG)**	Treatment delivery	GRP78	Radiation therapy, expression of therapeutic transgenes	Radiation + phage	GIRLPG peptide binds GRP78 and allows for adenovirus-mediated gene delivery to target tumor cells responding to radiation therapy	Enhances radiation therapy and therapeutic gene expression	Human cell lines, mouse xenograft models	No	(80)

**Table 3:** Interactions and signaling of GRP78-based treatments.

Mechanism	Interaction	Downstream signaling
**Direct GRP78 Binding**		
Epigallocatechin 3-gallate (EGCG)	Competitive inhibitor of the GRP78 NBD domain -> inhibits ATPase activity -> converts to inactive form	Interferes with anti-apoptotic function, prevents formation of anti-apoptotic GRP78-caspase7 complex
Honokiol	Binds to GRP78 NBD domain before post-translational folding	Interferes with folding of GRP78 into its active form
OSU-03012	Binds to GRP78 NBD domain before post-translational folding	Interferes with folding of GRP78 into its active form
HA15	Binds to GRP78 and inhibits its activity	Disinhibiting downstream elements (PERK, IRE1, and ATF6) -> triggers apoptotic and autophagic responses, triggers ER stress in GSCs
Anti-GRP78 antibody	Binds to surface GRP78	Interferes with surface GRP78 coreceptor functions -> disrupts the pro-survival PI3K/Akt/mTOR pathway
**Induction of ER Stress**		
Celecoxib and bortemozib	Celecoxib: Induction of ER stress through leakage of calcium from the ER, bortezomib: proteasome inhibitor	Higher expression of ER stress factors and induction of apoptosis
Perillyl alcohol (NEO100)	Induction of ER stress	Disrupts survival pathways -> reduction of invasive capacity
**Gene Therapy**		
RGD ligand-directed phage with GRP78 promoter	RGD tumor homing ligand and *GRP78* promoter used to target tumors	Delivery of therapeutic transgenes to tumors
TMZ-induced AAV phage with GRP78 promoter	RGD4C ligand binding, TMZ-induced GRP78 expression activates therapeutic genes with *GRP78* promoter	Delivery of suicide genes to tumors
GRP78-binding peptide (GIRLPG)	GIRLPG peptide binds GRP78 and allows for adenovirus-mediated gene delivery to target tumor cells responding to radiation therapy	Increased therapeutic gene expression -> increased therapeutic efficacy upon exposure to radiation
**Other**		
EGF-SubA	Cleavage of GRP78 at the hinge region connecting the ATPase and protein-binding domains	Suppression of function -> increased proteolytic activity and cytotoxicity
IT-139	Suppresses GRP78 (unknown mechanism)	Decreases GRP78 induction

### EGCG (Natural Product)

One natural product that has garnered particular attention is epigallocatechin 3-gallate (EGCG), a polyphenolic bioflavinoid from green tea extract (65). This compound is currently being investigated for its potential in enhancing chemotherapy. EGCG has been shown to increase sensitivity of GBM and multiple other tumor cells to various cytotoxic agents including 5-fluorouracil, taxol, vinblastine, gemcitabine, and tumor necrosis factor (TNF)-related apoptosis-inducing ligand (TRAIL) *in vitro*, as well as doxorubicin, paclitaxel, or interferon-α2b *in vivo*, using a preclinical model of intracranially injected GBM cells (58). UPR components are critical in mediating the effects of EGCG. EGCG binds and inactivates GRP78, interfering with its anti-apoptotic function (58). More specifically, EGCG acts as a competitive inhibitor, inhibiting ATPase activity and impairing GRP78 function by binding to the nucleotide-binding domain (NBD), an ATPase binding domain. Upon binding, EGCG also converts the GRP78 NBD domain from its active unfolded form to its inactive folded form. In addition, EGCG can prevent the formation of the anti-apoptotic GRP78-caspase7 complex (65). *In vitro* and *in vivo* studies demonstrated that EGCG enhanced the cytotoxic effects of TMZ when they were used simultaneously. However, for unclear reasons, when used as a monotherapy, EGCG failed to demonstrate significant antitumor activity (58). These results indicate that both GRP78 are involved in mediating the cytotoxic effects of EGCG and that this compound holds significant promise in improving response to TMZ.

### Honokiol (Natural Product)

Honokiol (HNK), a *Magnolia grandiflora* cell wall derivative, is another natural product of interest (66). Like EGCG, HNK was shown to preferentially bind to the unfolded form of the NBD of GRP78, with studies suggesting that HNK binds with a greater affinity than EGCG. Studies in neuroectodermal tumor cell lines, including GBM cell lines, showed that HNK induced apoptosis through ER stress with twice the efficacy of EGCG. In addition, HNK has shown efficacy in augmenting TMZ-induced damage in GBM tumor cells when used in combination (67). Furthermore, when used in TMZ-resistant GBM cell lines, honokiol alone successfully induced tumor cell death (68). HNK has also demonstrated synergistic effects when used in combination with fenretinide or bortezomib, which are ER stress inducers and antitumor agents (66). It has been suggested that HNK may interact with GRP78 before post-translational folding of newly synthesized GRP78, thus interfering with translation and reducing the ability for GRP78 to fold into its active form (66). Ultimately, studies have been limited to *in vitro* conditions, with future studies requiring more data from preclinical xenograft models.

### Cyclooxygenase Inhibitor-Based Therapies

Another agent of interest is OSU-03012, which was developed with a chemical backbone of celecoxib, a cyclooxygenase (COX2) inhibitor (69). OSU-03012 was shown to suppress GRP78 expression in GBM cells and to bind to the NBD of GRP78. Molecular docking and molecular dynamics studies demonstrated that, similarly to EGCG, OSU-03012 binding induced conformational changes, converting it from its active unfolded form to a more inactive folded form (65). However, EGCG binds with higher specificity to GRP78 than OSU-03012, and thus EGCG is thought to be a more effective inhibitor for GRP78 (81). Nevertheless, OSU-03012 has shown promise in sensitization of GBM tumor cells to radiotherapy. *In vitro*, pre-treatment of GBM cells with OSU-03012 enhanced radiosensitivity (69). *In vivo*, OSU-03012 sensitized tumor cells to radiotherapy and prolonged survival in GBM tumor mouse models (69). Variable expression of GRP78 can also affect cytotoxicity effects. Knockdown of GRP78 enhanced OSU-03012 lethality, while overexpression of GRP78 essentially eliminated its toxicity (69). In terms of mechanisms behind OSU-03012, other knockdown studies have determined that PERK signaling may mediate these effects. OSU-03012 is also an inhibitor of phosphoinositide-dependent kinase 1 (PDK1), a kinase that is important in signaling for growth factors (69).

An emerging alternative combination therapy based on celecoxib (Celebrex), which causes ER stress through leakage of calcium from the ER into the cytosol, combines it with GRP78 inhibition and the proteasome inhibitor bortezomib, which is known to trigger ER stress through accumulation of proteins (70). When celecoxib and bortezomib were used in combination, elevated expression of ER stress factors was detected and apoptotic cell death was greatly increased (70). Importantly, when celecoxib and bortezomib were used in conjunction with siRNA-mediated knockdown of GRP78, tumor cells were further sensitized to the treatment (70). A novel compound structurally similar to celecoxib, 2,5-dimethyl-celecoxib (DMC), when used with bortezomib instead of celecoxib, demonstrated the same effects but with higher potency (70). Furthermore, DMC alone has been shown to induce tumor-associated brain endothelial cell death through GRP78 and CHOP induction (82), suggesting that DMC is better drug of choice than celecoxib to be used in conjunction with bortezomib. Notably, clinical trials combining celecoxib with temozolomide have not demonstrated additional benefit (83). However, the above combination therapies, in conjunction with a GRP78-inhibiting agent, may hold promise for alternative future clinical trials.

### HA15 and IT-139

HA15 is a thiazole benzenesulfonamide small molecule inhibitor that directly interacts and targets GRP78, and has shown potential as a therapeutic agent in GBM. In melanoma cells, HA15 was shown to directly bind to GRP78, dissociating it from PERK, IRE1, and ATF6 and inhibiting its activity, which subsequently resulted in apoptotic and autophagic responses (74). In GBM, HA15 has similarly been shown to trigger ER stress in GSCs through GRP78-specific targeting (75). Another small molecular inhibitor, IT-139, has been shown to suppress GRP78 induction in therapy-resistant lung, prostate, liver, colon, pancreatic, gastric, and breast cancer cell lines, but not in non-cancerous cell lines, and decreased GRP78 in *in vivo* xenograft studies (76). Considering its efficacy in other cancer types, IT-139 could be a promising drug for GBM as well.

### EGF-SubA

A novel fusion protein called EGF-SubA has also demonstrated promise as a novel form of therapy that targets GRP78. This fusion protein was created by engineering epidermal growth factor (EGF) and the bacterial toxin SubA, which selectively cleaves GRP78 at a single site in the hinge region connecting the ATPase and protein-binding domains (77). EGF-SubA demonstrated tumor-specific proteolytic activity and cytotoxicity in GBM cell lines and enhanced sensitivity of cells to therapeutic doses of TMZ and ionizing radiation (77). In *in vivo* mouse models, EGF-SubA was also shown to delay tumor growth (77). Thus, this fusion protein also holds promise as monotherapy or combination therapy with TMZ and ionizing radiation.

### Antibody-Based

Antibody-based treatments have also emerged as therapeutic strategies. Studies have noted that antibodies targeting GRP78 obtain cytotoxic effect by interfering specifically with surface GRP78 coreceptor functions, disrupting the PI3K/Akt/mTOR signaling pro-survival pathway. Antibody binding therefore results in decreased tumor cell proliferation and colony formation, as well as enhanced apoptosis both *in vitro* and *in vivo* (45). Combining this anti-GRP78 antibody treatment with ionizing radiation therapy may have a sensitizing effect to radiation, with results showing more significant tumor growth delay with combination therapy (45). Importantly, it appears the GRP78 antibodies studied demonstrated specificity, binding specifically to cancer cells. Notably, antibody-based therapies must overcome challenges of chemotherapy delivery through the blood-brain barrier (BBB) in considerations of preclinical and clinical studies. In attempts to guide anti-GRP78 antibodies across the blood-brain barrier, antibody-conjugated nanoparticles have been shown to improve the accumulation of drugs in pathological sites and decrease side effects in normal tissue when utilized in neurodegenerative disorders (84). Future studies could also utilize this strategy with anti-GRP78 antibodies in GBM.

Another antibody based treatment, a micelle-based therapeutic delivery system, targets cell surface GRP78 limiting proliferation of GBM and GSC subpopulations (85). Micelles, “nanocarriers” for chemotherapeutics, were modified with two peptides. The first peptide, ^D^VAP, had high-affinity for GRP78 while the second, ^D^WSW, allowed blood-brain-barrier penetration necessary for *in vivo* access to tumors. Micelles were noted to co-localize with GRP78 on tumor cells. Subsequently, targeted micelles loaded with paclitaxel or parthenolide were noted to have potent anti-tumor activity, with increased survival of xenograft-bearing mice relative to free drug or non-targeted micelles. While such GRP78 targeted systems will require further study to ensure non-specific binding to csGRP in other systemic regions, results are promising.

### Gene Therapy

Phage-directed targeting of GRP78 for treatment delivery has generated significant interest from various labs due to the well-documented overexpression of GRP78 in aggressive tumors. A dual tumor-targeted phage, containing both a tumor homing ligand (the tripeptide Arg-Gly-Asp) and *GRP78* promoter, leverages tumor specificity of the bacteriophage with introduction of highly expressed GRP78 promoter within tumor cells. This was shown to be more effective in GRP78-guided expression of therapeutic transgenes compared to the standard cytomegalovirus promotor phage both *in vitro* and *in vivo* (78). Other attempts for delivery of therapeutic genes to GBM have utilized a hybrid AAV/phage to deliver a suicide genes under the control of a TMZ-induced promoter of *GRP78* (79). Dual tumor targeting is first accomplished when the phage capsid displays the RGD4C ligand that binds to an integrin receptor. The virus plasmid thus infects tumor xenografts in mice incorporating the viral plasmid within tumor cells. Subsequently, when TMZ is administered and GRP78 expression is upregulated in tumor tissue, the *GRP78* promoter is induced on the viral plasmid, which activates therapeutic gene expression (79). This method offers a compelling mode of combination therapy using TMZ and targeted suicide gene therapy that may potentially permit dose reductions of TMZ. Finally, another glioma-specific gene therapy study focused on direct targeting of cell surface GRP78. Specifically, radiation-induction of plasma membrane GRP78 on both tumor cells and associated endothelial cells was targeted by adenovirus. The authors utilized a GIRLPG peptide that specifically binds to GRP78 (80). The study found that using the GRP78-binding peptide resulted in increased gene expression in irradiated tumors after infection with the adenoviruses, demonstrating its increased efficacy in recognizing tumor cells that are responding to radiation therapy (80). While studies leveraging GRP78 for mediating treatment delivery in gene therapy are ongoing and must address targeting specificity and safety profiles in clinical delivery, ultimately, these therapies hold promise at supplementing current therapies.

### NEO100

Perillyl alcohol (POH), or NEO100, is another promising anti-cancer agent that has been shown to induce cytotoxicity through ER stress, as demonstrated by elevated expression of GRP78 (71). NEO100 is a monoterpene initially used as an oral treatment for systemic cancer (71). Clinical administration via intranasal delivery in patients has been successful in GBM, as a phase II trial in Brazil for intranasal NEO100 treatment of TMZ-resistant malignant gliomas was well-tolerated (72). NEO100 induces effects in GBM *via* disruption of survival pathways, with a reduction of invasive capacity of both chemosensitive and resistant glioma cell lines (71). These effects are likely mediated in part via ER stress and the UPR pathway, with administration of NEO 100 resulting in elevated levels of GRP78, CHOP, PARP, and ATF3 (71). Further, functional assessment via knockdown of GRP78 results in a significant decrease in tumor cell viability, with a corresponding increase in chemosensitivity. In parallel with the effects of GRP78 on GSCs noted above, NEO100 was also shown to be cytotoxic for different subtypes of GSCs (73). NEO100 may therefore offer a GRP-mediated treatment modality, currently in the clinical trial stage, that offers promising monotherapy or combination therapy with TMZ.

### Additional Treatments That Modulate GRP78 Without Direct Binding/Interaction

Various therapeutic strategies currently being investigated for efficacy in GBM tumors have notable effects on GRP78 expression without directly binding or interacting with the protein. The first of these therapies of interest is pterostilbene (PT). Pterostilbene is an analogue of resveratrol, a phenol compound found in plants (86). Studies have shown that when treated with PT, GSCs in GBM tumors demonstrated increased radiosensitivity (86). An increase of tumor suppressor miR-205 and negative modulation of GRP78 signaling suggested that these effects were mediated through the GRP78/miR-205 axis (86). Tubastatin A, a novel HDAC6 inhibitor, also functions through modulation of GRP78, resulting in reduced cell viability and induced apoptosis in TMZ-resistant glioma cells (87). Tubulin was noted to induce hyperacetylation of GRP78, resulting in dissociation of GRP78 from target proteins. Coupled with TMZ exposure, Tubulin A HDAC6 inhibition resulted in downstream effects that favored pro-apoptotic mechanisms (87).

GRP78 has been further indicated as a promising anti-angiogenic target, with studies noting that recombinant plasminogen kringle 5 (rK5) can induce apoptosis of brain derived dermal microvessel endothelial cells (MvEC) through mediation by GRP78 (88). Using knockdown studies, authors demonstrated that this apoptosis requires both GRP78 and lipoprotein receptor-related protein 1 (LRP1) (88). Of note, this study focused primarily on brain-derived MyECs, suggesting efficacy in GBM given a demonstration of increased GRP78 in GBM. Ultimately, further study would require direct evaluation of this treatment paradigm in GBM-derived endothelial cells. Finally, another treatment that involves GRP78 is the antimalarial agent chloroquine, which has also been shown to sensitize GBM cells to TMZ and whose effects were further enhanced by GRP78 knockdown (89).

### Therapies That Cause ER Stress

While GRP78 has been leveraged as a therapeutic target as described above, a large number of studies assess the efficacy of GBM treatments using GRP78 overexpression as an indicator of therapy-inducted ER stress. These treatments do not specifically upregulate GRP78, but rather overexpress GRP78 as a result of the ER stress that is caused upon treatment. While this GRP78 overexpression may mediate function of these treatments, or simply act an indicator of a naturally stressed tumor cell environment, further GRP78 specific studies would be required to clarify GRP78’s role in each of these therapies. These novel therapies, demonstrating GRP78 overexpression, include tumor necrosis factor-related apoptosis inducing ligand (TRAIL) (90), endothelial-monocyte activating polypeptide II (EMAP-II) (91), asiatic acid (92), ellagic acid (93), canavanine treatment under lack of arginine (94), C-150 (Mannich-type curcumin derivative) (95), gamitrinib with bromodomain and extraterminal (BET)-inhibitors (96), dihydroartemisinin (97), lysine demethylase KDM1A inhibitor (98), and withaferin A (99). More of these novel therapies are listed in **Table 4**. Perhaps the most compelling and widely implemented treatment modality demonstrating GRP78 elevation following treatment is tumor-treating fields (TTFields). TTFields is a recently FDA-approved antimitotic GBM treatment that acts *via* disruption GBM cell division and organelle assembly through low-intensity alternating electric fields (100). GRP78 was found to be elevated in cell lines following TTFields, indicating the induction of ER stress (101). Considering GRP78 overexpression and its role in tumor survival, GRP78 suppression in conjunction with these novel therapies may potentially improve and enhance their effects.

**Table 4:** Table of treatments that cause ER stress and result in GRP78 overexpression.

Treatment	Combination Therapy in GBM	Reference
**Pterostilbene**	Radiotherapy	(86)
**Tubastatin A**	TMZ	(87)
**Recombinant plasminogen kringle 5 (rK5)**	–	(88)
**Chloroquine**	TMZ + GRP78 knockdown	(89)
**Tumor necrosis factor-related apoptosis inducing ligand (TRAIL)**	–	(90)
**Endothelial-monocyte activating polypeptide II (EMAP-II)**	–	(91)
**Asiatic acid**	–	(92)
**Ellagic acid**	–	(93)
**Canavanine treatment under lack of arginine**	–	(94)
**C-150 (Mannich-type curcumin derivative)**	–	(95)
**Gamitrinib with bromodomain and extraterminal (BET)-inhibitors**	–	(96)
**Dihydroartemisinin**	–	(97)
**Lysine demethylase KDM1A inhibitor**	–	(98)
**Withaferin A**	–	(99)
**Tumor treating fields (TTFields)**	–	(100, 101)
**RDC11 (ruthenium derived compound)**	–	(15, 102)
**Terpyridineplatinum (II) complexes**	–	(15, 103)
**2-deoxy-D-glucose + cisplatin**	–	(15, 104)
**SKI-II (4- ((4-(4-chlorophenyl)-2-thiazolyl)amino)phenol)**	TMZ	(15, 105)
**5-androstene 3β,17α diol (17α-AED)**	–	(15, 106)
**Berberine**	–	(15, 107, 108)
**Bufalin**	–	(15, 109)
**Copper (Cu)**	–	(15, 110)
**Glucosamine**	–	(15, 111)
**Nelifnavir/atazanavir**	–	(10, 15, 112)
**Minocycline (7-dimethylamino-6-desoxytetracycline Mino);**	–	(15, 113)
**Phenethyl isothiocyanate**	–	(114–116)
**Prenyl-phloroglucinol derivative [2,4-bis (4-fluorophenylacetyl)phloroglucinol]**	–	(15, 117)
**S1 (BH3 mimetics)**	–	(15, 118)
**Schweinfurthin analogs**	–	(15, 119)
**Sulindac sulfide**	–	(15, 120)
**Unsaturated fatty acids**	Radiotherapy	(15, 121)
**Valproate**	–	(15, 122, 123)
**Wogonin**	–	(15, 124)
**Carbamazepine**	–	(15, 125)
**Cyclosporine A**	–	(15, 126, 127)
**Ethanol**	–	(15, 128, 129)
**Lead (Pb acetate)**	–	(15, 130, 131)
**Mercury (HgCl2)**	–	(15, 130)
**Oleyl glucosaminide derivative**	–	(15, 132)
**Sesquiterpene coumarin DAW22**	–	(15, 133)

Overall, GRP78 can be leveraged in many diverse ways and through various approaches to improve GBM therapy, demonstrating great potential as a therapeutic target in improving first-line therapies as well as in developing alternative therapies. Ultimately, further study of GRP78 and UPR targeting in GBM is warranted. In addition to the future studies suggested throughout this review, studies must clarify GRP-specific and GBM-specific targeting that crosses the blood brain barrier, minimize effects on normal non-cancerous cells within the host, and maximize an acceptable safety profile for a very promising target.

## Conclusion

GRP78 and other components of the UPR have important roles in mediating GBM-specific survival, therapeutic resistance, and tumor progression. Many treatment modalities targeting the UPR and GRP78 are under investigation, with some at the clinical trial stage. Many of these therapies sensitize tumor cells to the current standard-of-care therapies, TMZ and radiation therapy, suggesting potential for combination therapy with GRP78-suppressing agents and current therapy. Further investigation is warranted to evaluate the efficacy of these treatment modalities within the clinic, as well as synergistic effects in the cases involving combination therapies. While GBM remains a devastating disease, GRP78-based therapies may hold significant promise in prolonging overall survival, delaying tumor progression, and decreasing treatment resistance and recurrence in GBM, ultimately providing hope that future treatments might convert GBM from a malignant disease to a chronic one.

## Author Contributions

KL did the majority of the literature search and wrote the majority of the manuscript. KL also designed the figures and made the tables. KT made edits to the manuscript, gave crucial feedback for the text, and offered significant guidance in the design of the figures. FA is the principal investigator. FA gave guidance on the focus and general direction of the paper, provided detailed edits, added specific data based on additional literature search, and did much of the rewriting. All authors contributed to the article and approved the submitted version.

## Funding

Laboratory studies evaluating noncoding RNAs modulating the glioblastoma cell stress response is funded by the Margaret E. Early Medical Research Trust. Laboratory work and publication fees are also supported by the USC Department of Neurosurgery Research Startup Fund.

## Conflict of Interest

The authors declare that the research was conducted in the absence of any commercial or financial relationships that could be construed as a potential conflict of interest.
